# Guidelines for the diagnosis and treatment of neuroangiostrongyliasis: updated recommendations

**DOI:** 10.1017/S0031182020001262

**Published:** 2021-02

**Authors:** Vernon Ansdell, Kenton J. Kramer, Jourdan K. McMillan, William L. Gosnell, Gerald S. Murphy, B C Meyer, Elizabeth U. Blalock, Johnnie Yates, Louis Lteif, Olivia A. Smith, Marian Melish

**Affiliations:** 1 Department of Tropical Medicine, Medical Microbiology and Pharmacology, John A Burns School of Medicine, University of Hawaii at Manoa, Honolulu, HI, USA; 2 East Maui Community Medicine, Hana, HI; 3 VA Pacific Islands Health Care System, Honolulu, HI, USA; 4 Hawaii Permanente Medical Group, Honolulu, HI, USA; 5 Sharp Healthcare, San Diego, CA, USA; 6 Department of Pediatrics, John A Burns School of Medicine, University of Hawaii at Manoa, Honolulu, HI, USA

**Keywords:** Neuroangiostrongyliais, Diagnosis, Treatment

## Abstract

A subcommittee of the Hawaii Governor's Joint Task Force on Rat Lungworm Disease developed preliminary guidelines for the diagnosis and treatment of neuroangiostrongyliasis (NAS) in 2018 (Guidelines, 2018). This paper reviews the main points of those guidelines and provides updates in areas where our understanding of the disease has increased. The diagnosis of NAS is described, including confirmation of infection by real-time polymerase chain reaction (RTi-PCR) to detect parasite DNA in the central nervous system (CNS). The treatment literature is reviewed with recommendations for the use of corticosteroids and the anthelminthic drug albendazole. Long-term sequelae of NAS are discussed and recommendations for future research are proposed.

## Introduction

Neuroangiostrongyliasis is a parasitic infection of the central nervous system caused by larvae of the rat lungworm, *Angiostrongylus cantonensi*s. Infection may cause a spectrum of disease ranging from mild, self-limited headache to severe, neurologic debilitation, coma and rarely death. Diagnosis is often delayed due to its rarity as well as its protean and often unusual or potentially confusing clinical manifestations.

Rats in the genus *Rattus* are the definitive host for *A. cantonensis* and numerous species of gastropods serve as the intermediate host (Cowie, 2013*a*). Infection occurs after ingestion of gastropods or paratenic hosts containing viable larvae. Within the definitive host, a transient 4-week period in the CNS is needed before the parasite migrates to the pulmonary arteries (Jindrák, 1968). In humans, the parasite enters the CNS, resulting in meningitis, encephalitis or radiculomyelitis.

A subcommittee of the Hawaii Governor's Joint Task Force on Rat Lungworm Disease developed preliminary guidelines for the diagnosis and treatment of NAS (Guidelines, 2018). This paper reviews the main points of those guidelines and updates them in areas where our understanding of the diagnosis, treatment and sequelae of NAS has expanded.

## Diagnosis

A presumptive diagnosis of NAS can be made on clinical grounds based on the following:

• Characteristic symptoms and signs.
• An exposure history, which includes residence in or travels to an endemic area.
• Eosinophilic meningitis (EOM), diagnosed by lumbar puncture (LP) and analysis of cerebrospinal fluid (CSF).

A definite history of contact with or ingestion of an intermediate or paratenic host is not necessary to make a diagnosis of NAS and initiate treatment. A presumptive diagnosis based on the criteria listed above should be considered a sufficient basis to begin treatment. The diagnosis is definitively established when *A. cantonensis* larvae are seen in the CSF (or eye) but this is uncommon (Kuberski and Wallace, 1979). Therefore, a positive RTi-PCR test for *A. cantonensis* DNA in the CSF is valuable for confirmation of NAS (Qvarnstrom *et al*., 2016), although it is not necessary prior to starting treatment provided that EOM has been documented, there is a potential exposure history, and there are no other obvious causes of the illness.

### Exposure history

An exposure history (including a thorough food, beverage and travel history) should be elicited. This history should include:

• Ingestion of raw or undercooked snails or slugs (intermediate hosts), either intentional or unintentional.
• Eating uncooked, unwashed or inadequately washed vegetables or fruits.
• Eating raw or undercooked paratenic hosts.
• Consumption of potentially contaminated beverages (raw, blended vegetable juice) (Tsai *et al*., 2004).
• Contact with snails or slugs
• Residence in an endemic area.
• Recent travel to an endemic area.

Children, particularly those under 5 years of age or those with developmental disabilities, may be at higher risk of exposure because of an increased tendency to handle or ingest slugs or snails (Wan and Weng, 2004; Cowie, 2013*b*). Some cases in adults and older children have resulted from intentionally swallowing snails or slugs on a dare, and these patients may be reluctant to divulge the exposure (New *et al*., 1995; Murphy and Johnson, 2013). Despite a careful food history, definite exposure is often not identified. Therefore, residence in an endemic area should be considered a sufficient risk factor for exposure to *A. cantonensis*.

A history of travel to endemic areas should also be sought. NAS has been reported from tropical and subtropical regions of Southeast Asia, China, Taiwan, Australia, islands of the North and South Pacific, India, Sri Lanka, the Caribbean, South America, southern portions of the USA and some countries in Africa (Ansdell and Wattanagoon, 2018; Barratt *et al*., 2016; Federspiel *et al*., 2020). There is evidence that the parasite's geographical range may be changing due to multiple factors including climate change (Rosenthal, 2009). Thus, travel to an endemic region within an appropriate time frame is also a risk factor (York *et al*., 2015).

The date of exposure should be identified whenever possible as this may influence treatment. The median time from exposure to presentation is typically between 1 and 3 weeks, although the incubation period of NAS can range from a few days to more than 6 weeks (Yii, 1976; Wang *et al*., 2008; Graeff-Teixeira *et al*., 2009).

### Characteristic symptoms

There is a broad spectrum of clinical presentation of NAS. Early in the course of illness patients may have nonspecific symptoms and signs, and the clinical manifestations may evolve over days to weeks to include more specific symptoms (Murphy and Johnson, 2013).

A gastrointestinal prodrome of nausea, abdominal pain, diarrhoea and vomiting may manifest within hours to a few days of ingestion of an infected intermediate host. There may also be malaise, low-grade fever, cough, pruritus and rash (Cross, 1978). This prodrome is usually self-limited and results from penetration of the intestinal wall by infectious larvae that gain access to the circulation and migrate to other organs en route to the CNS (Yii, 1976; Kwon *et al*., 2013). There may be a subsequent asymptomatic period lasting days to weeks, followed by the appearance of headache and other neurologic signs and symptoms (Graeff-Teixeira *et al*., 2009; Martins *et al*., 2015).

General but nonspecific symptoms include headache, feeling feverish, nausea, vomiting, photophobia, insomnia, anxiety, fatigue and lethargy (Cross, 1978). More suggestive symptoms include a new, severe and unremitting headache (Chau *et al*., 2003), migratory myalgias, non-dermatomal sensory symptoms (paresthesia and hyperesthesia, frequently described as pruritus, pain, tingling, crawling or burning sensations), diplopia, limb weakness, bowel or bladder dysfunction and seizures (Hsu *et al*., 2009; Wang *et al*., 2008; Martins *et al*., 2015). Children tend to present with fever, irritability, aversion to touch or being held, somnolence, gastrointestinal symptoms (vomiting, poor appetite, nonspecific abdominal pain), muscle twitching, seizures and weakness of the extremities (Hwang and Chen, 1991).

### Signs on physical examination

A complete neurologic examination including an ophthalmological examination for extraocular muscle function, papilledema and the presence of larvae, should be performed in all patients suspected of having NAS. Suggestive physical examination finding includes fever, meningismus, weakness, non-dermatomal sensory abnormalities, cranial nerve (CN) deficits (especially palsies of the abducens nerve (CN VI) causing diplopia, facial nerve (CN VII) causing hemifacial weakness or auditory nerve (CN VIII) resulting in tinnitus or hearing loss), tremors, inability to coordinate fine motor movements, ataxia and altered level of consciousness (Punyagupta *et al*., 1990; Murphy and Johnson, 2013; Sawanyawisuth *et al*., 2013).

#### Lumbar puncture

The hallmark of NAS is EOM. Therefore, once NAS is suspected a LP and examination of the CSF is required to make a diagnosis of EOM (Kuberski and Wallace, 1979; Wang *et al*., 2008; Sawanyawisuth *et al*., 2013). Typical LP and CSF findings in NAS include an elevated opening pressure and increased white blood cells, particularly eosinophils. It is important to record the opening CSF pressure because elevated intracranial pressure (ICP) likely contributes to some of the neurologic damage of NAS and an increased ICP should prompt removal of a large volume of CSF (e.g. 20–40 mL in adults) which often results in dramatic (but temporary) relief of headache. Relief of headache with the initial LP may suggest that subsequent LPs could be beneficial if severe headaches return or if there is neurologic deterioration (Sawanyawisuth *et al*., 2013).

EOM has been defined as the presence of 10 or more eosinophils *μ*L^−1^ of CSF and/or eosinophils accounting for more than 10% of the white blood cells when there are at least 6 total WBC *μ*L^−1^ in the CSF (Kuberski and Wallace, 1979). However, eosinophils may be few or even absent early in the disease (Kuberski and Wallace, 1979; Schmutzhard *et al*., 1988) and the presence of any eosinophils in the CSF should be considered abnormal. A repeat LP several days after the initial test may be indicated if clinical suspicion of NAS remains high. Nearly all patients will have a CSF eosinophilic pleocytosis at some point during the course of their illness (Wang *et al*., 2008). Clinicians should verify that the laboratory searches for eosinophils in the CSF using appropriate stains such as Giemsa or Wright. Routine CSF studies to exclude other causes of meningitis should also be ordered. In cases where there are a strong exposure history and convincing symptoms and signs, it may be appropriate to start treatment for NAS even if the strict criteria for the diagnosis of EOM have not been met (i.e. if there are <10 eosinophils *μ*L^−1^ in the CSF).

### Real-Time polymerase chain reaction (RTi-PCR) test for confirmation of NAS

RTi-PCR for *A. cantonensis* DNA in the CSF should be used to confirm the diagnosis of NAS if the test is available and larvae are not identified in the CSF (Qvarnstrom *et al*., 2016). In some instances, RTi-PCR may be negative early in the disease but then become positive later. If clinical suspicion for NAS is high but an initial RTi-PCR is negative, the LP should be repeated in approximately 5–10 days. It is important to note that it is not necessary to wait for RTi-PCR results before initiating treatment.

### Additional laboratory testing

A complete blood count with differential should be performed and the absolute eosinophil count should be calculated to establish the presence of significant eosinophilia. Peripheral eosinophilia (⩾ 500 cells *μ*L^−1^) is often present during the course of illness but may be absent (Sawanyawisuth *et al*., 2013). Thus, eosinophilia in the blood is suggestive but not diagnostic of NAS. Serological testing for acute infection is not recommended because interpretation is difficult and seroconversion may take several weeks (Murphy and Johnson, 2013).

### Diagnostic imaging

There are no pathognomonic radiographic findings of NAS but magnetic resonance imaging (MRI) of the brain may demonstrate the following: leptomeningeal enhancement in post-contrast studies; increased signal intensity in the subcortical white matter on T2 weighted and FLAIR images; and nodular, linear or hockey stick-like lesions in the white matter on gadolinium-enhanced T1 images (Lai *et al*., 2007; Tsai *et al*., 2011; Yang *et al*., 2019; McAuliffe *et al*., 2019). Brain MRI may also be normal during the first few weeks of illness. Newer MRI modalities, such as 3D MRI, may offer more specific findings but this requires further study. In patients with myeloradicular symptoms and signs, MRI of the spine is recommended (Lai *et al*., 2007; Diao *et al*., 2011; McAuliffe *et al*., 2019).

Computerized tomography (CT) scans of the brain have not been shown to be useful in diagnosing NAS (Martins *et al*., 2015). However, some case reports have noted nodular lesions in chest CTs of patients, presumably caused by migratory larvae or young adults (Cui *et al*., 2011). *Angiostrongylus cantonensis* is a neurotropic parasite and primarily presents with neurologic symptoms in humans, but pulmonary involvement may be underrecognized and adult *A. cantonensis* has been reported in the pulmonary arteries at autopsy (Prociv and Turner, 2018). Therefore, a chest CT should be considered if respiratory symptoms are present.

### Differential diagnosis

In Hawaii, *A. cantonensis* is the leading cause of infectious EOM (Hughes *et al*., 2003; Hochberg *et al*., 2007, 2011). However, the differential diagnosis includes other infectious diseases, many of which are geographically restricted but should be considered when there is an appropriate exposure history. These include cerebral gnathostomiasis, neurococcidioidomycosis, neurocysticercosis, cerebral schistosomiasis, baylisascariasis, visceral toxocariasis, cerebral paragonimiasis and neurotrichinosis. Noninfectious causes of EOM include ventriculoperitoneal shunts, lymphomas, medications (e.g. ibuprofen, ciprofloxacin) and intrathecal contrast material (Lo Re III and Gluckman, 2001).

## Clinical management of neuroangiostrongyliasis

### Initial and serial lumbar punctures

LPs can be therapeutic as well as diagnostic (Sawanyawisuth and Sawanyawisuth, 2008). Assuming there are no contraindications, LPs may be repeated as often as necessary to relieve headaches. Serial LPs may be the best treatment option for patients in whom high dose corticosteroids are contraindicated or poorly tolerated.

### High dose corticosteroids

It is recommended that high dose corticosteroids be used in most patients with NAS, although they may be unnecessary in very mild cases (Sawanyawisuth and Sawanyawisuth, 2008). They should be started as soon as a presumptive diagnosis is made, i.e. before RTi-PCR confirmation is available.

Corticosteroids such as prednisolone or prednisone (60 mg day^−1^ in adults or 1–1.5 mg kg^−1^ day^−1^ in children in divided doses not to exceed 80–100 mg) or dexamethasone (10–20 mg kg^−1^ day^−1^ in adults and 0.6 mg kg^−1^ day^−1^ in children in divided doses) improve headache caused by NAS. A double-blind, randomized, controlled trial in Thailand studied prednisolone 60 mg day^−1^ for 14 days *vs* placebo in 129 patients over 15 years of age with EOM (Chotmongkol *et al*., 2000). Randomization to achieve equal severity between groups was conducted and patients with altered consciousness were excluded. The primary endpoint was the number of patients who still had a headache after 14 days and secondarily, duration of the headache. The number of repeat LPs performed to relieve headache was also noted. There were 5 patients in the prednisolone group with a headache at day 14 compared to 25 in the placebo group, *P* < 0.0001. Patients in the prednisolone group had a median time to complete resolution of the headache of 5 days compared to 13 days in the placebo group, *P* < 0.0001. There were 7 repeat LPs in the prednisolone group *vs* 22 in the placebo group, *P* = 0.002. Serious side-effects were not noted. The exclusion of patients with severe disease prevents the generalizability of the results to patients with altered mental status. The investigators reported no relapses during follow up, which suggests that the patient population may have had relatively mild disease.

A retrospective study in Thailand (Chotmongkol and Sawanyawisuth, 2002) examined the role of high dose corticosteroids in a group of 7 patients with severe meningoencephalitis, all of whom were critically ill and in coma. In this study, corticosteroids did not appear to be of benefit in comatose patients. Six patients died and one remained in coma. It is unclear if corticosteroids might have been useful if the patients had been treated earlier in the course of their disease.

Corticosteroids are typically given for 2 weeks but may need to be tapered over several weeks or months. A study in Thailand assessed the effectiveness of a 1-week course of prednisolone 60 mg day^−1^ in 52 patients (Sawanyawisuth *et al*., 2004). Eight patients (15%) relapsed after corticosteroids were discontinued and 6 of them had to either resume steroids or undergo additional LPs to relieve headaches. It is possible that shorter courses of corticosteroids may be effective in relatively mild cases or they can be used in combination with repeated LPs in patients who do not tolerate longer courses of corticosteroids.

It remains to be seen whether corticosteroids improve other important outcomes such as long-term disability and death. Large, double-blind, controlled trials are needed to resolve this issue.

## Treatment with anthelminthics

Several anthelminthic drugs have been used to treat NAS, including benzimidazoles such as albendazole, mebendazole, thiabendazole and fenbendazole. Other anthelminthic drugs such as ivermectin and levamisole have also been used in some countries. Of the benzimidazoles, albendazole appears to be the best option for treating NAS. The drug crosses the blood-brain barrier and results in relatively high levels in the CSF compared to other benzimidazoles. Absorption is improved up to 5-fold if it is taken with a fatty meal (Bloom and Ryan, 2013).

There is only one double-blind, placebo-controlled, randomized trial of an anthelminthic without corticosteroids in neuroangiostrongyliasis (Jitpimolmard *et al*., 2007). Albendazole (15 mg kg^−1^ day^−1^ in 2 divided doses) or placebo was given for 2 weeks to Thai patients with NAS. The primary outcome was the number of patients with headache after 2 weeks, and secondary outcomes were the duration to the resolution of headache in days, acetaminophen use, and the number of LPs. Seven of 34 patients (21%) in the albendazole group still had a headache after 14 days compared to 13/32 (41%) in the placebo arm, *P* = 0.08. Mean duration of the headache was 9 days in the albendazole arm *vs* 16 in the placebo arm, *P* = 0.05. Mean tablets of acetaminophen used were 24 with albendazole *vs* 38 with placebo, *P* = 0.01. The number of LPs was not significantly different. There was no indication that patients on albendazole fared worse than those who received placebo. This well-designed trial suggested that albendazole alone may be effective in treating NAS patients who do not have altered consciousness, but the outcomes barely achieved statistical significance.

There are few case studies where anthelminthics were employed in all or some of the patients. Hwang and Chen reported on a case series of 82 children in Taiwan with neuroangiostrongyliasis (Hwang and Chen, 1991). Of the total, 83% of these children had eaten *Lissachatina fulica* snails so they may have had high inocula of infective larvae. Worms were recovered from the CSF in 41.5% of cases, also suggesting a high worm burden. Although it was not designed as a treatment trial, patients were initially treated with glycerol, mannitol or steroids, along with LPs. Late in the study, anthelminthics (levamisole 2.5 mg kg^−1^ day^−1^ or albendazole 10 mg kg^−1^ day^−1^, for 3 weeks) were given in place of steroids to 22 children. There were no complications or adverse sequelae in this subgroup, despite 4 deaths (along with 6 children who developed long-term sequelae) in the steroid group. The 4 deaths were all in patients who were comatose at admission. This study suggests that anthelminthics were not detrimental in children with NAS, although there is not enough evidence to deduce efficacy. Nonetheless, it provided further evidence that comatose patients with NAS do not benefit from corticosteroids (Chotmongkol and Sawanyawisuth, 2002).

A commonly reported dosage of albendazole in the treatment of NAS is 15 mg /kg^−1^ day^−1^ in two divided doses for 14 days (Jitpimolmard *et al*., 2007). Albendazole is well-tolerated, but because of rare idiosyncratic reactions including liver failure and pancytopenia, monitoring of the CBC with differential and liver enzymes is recommended. The drug is teratogenic in some animals and is listed as pregnancy category C (Bloom and Ryan, 2013); therefore, it should be used with caution in pregnant women particularly in the first trimester.

Animal studies have shown that anthelminthics are most effective against young larvae and need to be given within the first 1–3 weeks after infection to be beneficial. An animal study found albendazole to be most effective when given 5–14 days after infection, after which efficacy in killing larvae rapidly decreased (Hwang and Chen, 1988). These data inform the recommendation for starting anthelminthic therapy as soon as a presumptive diagnosis is made rather than waiting for confirmation with RTi-PCR. Theoretical benefits of killing the larvae early in the course of the illness include limiting the damage caused by larval migration and preventing smaller larvae from maturing into larger larvae that can cause more destruction. As *A. cantonensis* larvae mature in the CNS, their volume increases over 1000 times (Prociv and Turner, 2018).

Data on the use of both anthelminthics and corticosteroids in the treatment of NAS have not shown the superiority of the combination but suggest that it is well-tolerated and safe. An uncontrolled study that evaluated 41 patients with EOM who were treated with prednisolone 60 mg day^−1^ and mebendazole 10 mg kg^−1^ day^−1^ for 2 weeks showed resolution of headaches in 37 patients (90%) after the 2-week course of treatment (Chotmongkol *et al*., 2006). The median length of time until complete disappearance of headaches was 3 days. Serious side-effects were not observed (Chotmongkol *et al*., 2006). The authors concluded that the combination of prednisolone and mebendazole for 2 weeks was safe and effective in relieving headaches in patients with EOM.

In an unblinded treatment trial comparing prednisolone plus albendazole with prednisolone alone for the treatment of EOM (Chotmongkol *et al*., 2009), no significant differences in the number of patients who still had headaches after 14 days or in the median duration of headaches were found. However, the patients in this study appeared to have mild disease and there were differences in the baseline characteristics between the group, notably the albendazole group had a longer duration of symptoms (13.5 days) as compared to the control group (7 days).

The use of anthelminthic drugs to treat NAS has been controversial for decades because of theoretical concerns that rapid larval death might exaggerate the inflammatory response in the brain and spinal cord (Bowden, 1981; Pien and Pien, 1999; Leone *et al*., 2007; Wang *et al*., 2008). However, these concerns have not been definitively supported by either clinical or animal studies. In fact, animal studies have shown a decrease in inflammation when albendazole alone is used for treatment (Lan *et al*., 2004). We believe that in most cases of NAS the potential benefits of albendazole outweigh the theoretical risks. The decision to use anthelminthics, however, should be made on a case-by-case basis and factors such as time of exposure (if known) should be considered. Until definitive safety data are available, we recommend that corticosteroids be given concurrently with albendazole when used to treat NAS.

### Patient monitoring

Patients with NAS should be followed closely with serial neurologic exams. Worsening symptoms or signs should prompt neuroimaging (e.g. brain or spinal cord MRI) and possibly a repeat LP. If the ICP is high, removal of large volumes of CSF may be indicated. New blood pressure elevations may herald an increased ICP (Cushing reflex). Albendazole may cause an elevation in liver transaminases and monitoring is recommended. If corticosteroids are used for more than 14 days (or if they are used in children), the dosage should be carefully tapered due to the potential for adrenal suppression (Williams, 2018).

### Long-term sequelae

Anecdotal experience in Hawaii has shown that many patients develop chronic neurologic sequelae that significantly impact their functional status and quality of life. One case study (Hochberg *et al*., 2011) reported that headaches, paresthesias, hyperesthesias and numbness could persist for months. In addition, an unpublished study of 10 individuals diagnosed with NAS in Hawaii found that a significant number reported residual symptoms (e.g. paresthesias, neuropathic pain, myalgias and sleep disturbances) that continued years after the acute infection.

### Pain management

#### Acute phase pain management.

Large volume (e.g. 20–40 mL in adults) LP is effective in relieving the acute headache associated with NAS (Murphy and Johnson, 2013). High dose corticosteroids may also reduce morbidity due to headaches. Nonsteroidal anti-inflammatory drugs (NSAIDs) may be relatively contraindicated because of the increased risk of gastrointestinal bleeding if high dose steroids are concurrently given. NSAIDs may also increase the risk of intracranial bleeding. Opioids should be used judiciously (if at all) because they may cause altered mental status (e.g. confusion, sedation) and because of their potential for abuse.

#### Treatment of chronic sequelae

Chronic neuropathic pain can occur as a consequence of NAS. Gabapentin, pregabalin, tricyclic antidepressants, selective serotonin reuptake inhibitors, serotonin-norepinephrine reuptake inhibitors and ketamine have all been used with variable success to treat chronic neuropathic pain due to other causes (Cruccu and Truini, 2017). A review of multiple randomized controlled trials on pharmacotherapy for neuropathic pain in adults (Finnerup *et al*., 2015) concluded that limited efficacy, large placebo responses, inadequate diagnostic criteria and poor phenotypic profiling accounted for modest trial outcomes.

Most trials on the use of cannabinoids for neuropathic pain showed no demonstrable effect (Aviram and Samuelly-Leichtag, 2017). Meng *et al* similarly evaluated 11 RCTs and concluded that there might be a significant, but clinically small, reduction in mean pain scores but the studies were not of consistently high quality (Meng *et al*., 2017).

A Cochrane review of acupuncture for the relief of neuropathic pain in adults (Ju *et al*., 2017) found ‘insufficient evidence to support or refute the use of acupuncture for neuropathic pain in general, or for any specific neuropathic pain condition when compared with sham acupuncture or other active therapies.’

Persons with chronic symptoms related to NAS may benefit from a multidisciplinary approach that includes physical and occupational therapy, psychosocial support and possibly alternative/complementary therapies. Neurology, physiatry and pain management consultation may also be helpful. In Hawaii, patient support groups enable patients, caregivers and healthcare workers to share information and experiences, and may help patients cope with the long-term physical and psychological effects of their infection.

## Discussion

The spectrum of disease caused by *A. cantonensis* in Hawaii reflects that reported from other regions (Oehler *et al*., 2014). However, serious illness appears more common in Hawaii and countries such as Australia and Jamaica (Blair *et al*., 2013; Evans-Gilbert *et al*., 2014) compared to Thailand, where most of the treatment trials have been conducted. This difference may be due to the inoculum of infectious larvae ingested. In Thailand, aquatic snails and paratenic hosts (which account for the majority of infections) are believed to have relatively lower larval burdens (Eamsobhana, 2014). In contrast, terrestrial gastropods in Hawaii (which are intentionally or inadvertently ingested) may contain enormous larval loads. The disproportionate amount of severe disease in Hawaii and countries such as Australia emphasizes the importance of early diagnosis and treatment of NAS, prior to the development of serious CNS damage.

NAS poses a significant diagnostic challenge. The gold standard finds the larvae in the CSF or eye but this is rare. Various serological assays have been developed in international reference laboratories (Wilkins *et al*., 2013); however, none have been validated in the USA. Due to high specificity, RTi-PCR is becoming the gold standard for confirming active infection.

At present, NAS patients are best managed with LPs to reduce ICP and provide headache relief, high-dose corticosteroids to reduce inflammation in the CNS and albendazole to minimize damage from migrating parasites. It should be noted that the effectiveness of albendazole depends on the parasite's stage of development in the CNS. Based on animal studies, maximum parasite killing occurs during periods of rapid growth, which is 5–14 days after ingestion (Prociv and Turner, 2018). Because parasite death may increase inflammation, albendazole should be given under steroid coverage in symptomatic patients.

## Future directions

Because of the limited availability of data on human NAS, well designed, randomized, double-blind, placebo-controlled trials are needed to resolve many of the questions about the diagnosis and treatment. Such studies will probably require multi-centre international collaboration. Diagnostic delay is common in NAS and there is evidence that early treatment may improve outcomes. Therefore, future research should seek to improve early diagnosis as well as define the treatment window for anthelminthic use. More sensitive molecular tests for CSF, blood and urine could help clinicians make the diagnosis earlier and potentially less invasively, thereby reducing morbidity and long-term sequelae. As MRI is being increasingly used in the diagnosis of NAS, an updated analysis of MRI findings in NAS patients would be valuable. In addition, newer, more sophisticated MRI modalities may provide earlier and more specific diagnostic clues and may even provide information on clinical response to treatment.
